# MoS_2_-ZnO Nanocomposite Mediated Immunosensor for Non-Invasive Electrochemical Detection of IL8 Oral Tumor Biomarker

**DOI:** 10.3390/diagnostics13081464

**Published:** 2023-04-18

**Authors:** Cittrarasu Vetrivel, Ganesan Sivarasan, Kaliannan Durairaj, Chinnasamy Ragavendran, Chinnaperumal Kamaraj, Sankar Karthika, Huang-Mu Lo

**Affiliations:** 1 Carbon Capture Lab, Department of Chemical Engineering, SSN College of Engineering Kalavakkam, Chennai 603110, Tamil Nadu, India; 2 Department of Anatomy, Saveetha Dental College and Hospitals, Saveetha Institute of Medical and Technical Sciences, Chennai 600077, Tamil Nadu, India; 3 Department of Environmental Engineering and Management, Chaoyang University of Technology, Taichung 41349, Taiwan; 4 Zoonosis Research Center, Department of Infection Biology, School of Medicine, Wonkwang University, Iksan 54538, Republic of Korea; 5 Department of Conservative Dentistry and Endodontics, Saveetha Dental College and Hospitals, Saveetha Institute of Medical and Technical Sciences, Chennai 600077, Tamil Nadu, India; 6 Interdisciplinary Institute of Indian System of Medicine (IIISM), Directorate of Research and Virtual Education, SRM Institute of Science and Technology (SRMIST), Kattankulathur 603203, Tamil Nadu, India; 7 Department of Biotechnology, Mahendra Arts and Science College (Autonomous), Namakkal 637501, Tamil Nadu, India

**Keywords:** immunosensor, molybdenum disulfide, zinc oxide nanoparticles, biomarkers, electrochemical biosensor, interleukin-8, oral cancer

## Abstract

In order to support biomolecule attachment, an effective electrochemical transducer matrix for biosensing devices needs to have many specialized properties, including quick electron transfer, stability, high surface area, biocompatibility, and the presence of particular functional groups. Enzyme-linked immunosorbent assays, gel electrophoresis, mass spectrometry, fluorescence spectroscopy, and surface-enhanced Raman spectroscopy are common techniques used to assess biomarkers. Even though these techniques provide precise and trustworthy results, they cannot replace clinical applications because of factors such as detection time, sample amount, sensitivity, equipment expense, and the need for highly skilled individuals. For the very sensitive and targeted electrochemical detection of the salivary oral cancer biomarker IL8, we have created a flower-structured molybdenum disulfide-decorated zinc oxide composite on GCE (interleu-kin-8). This immunosensor shows very fast detection; the limit of detection (LOD) for interleukin-8 (IL8) detection in a 0.1 M phosphate buffer solution (PBS) was discovered to be 11.6 fM, while the MoS_2_/ZnO nanocomposite modified glassy carbon electrode (GCE) demonstrated a high catalytic current linearly from 500 pg to 4500 pg mL^−1^ interleukin-8 (IL8). Therefore, the proposed biosensor exhibits excellent stability, high accuracy sensitivity, repeatability, and reproducibility and shows the acceptable fabrication of the electrochemical biosensors to detect the ACh in real sample analysis.

## 1. Introduction

Cancer is the second most common cause of death around the world; about 8.8 million deaths reported in 2015 were due to cancer. Oral squamous cell carcinoma (OSCC) is the 11th most common cancer worldwide; it accounts for more than 90% of total oral cancer cases. Cancer is abnormal and uncontrolled cell growth [1,2]. Oral cancer (OC) disease is the 6th most demise-causing malignant growth and can happen in lips, cheeks, gingiva, or taste buds in the mouths of people [3]. Genome-based cancer biomarkers are applied in a variety of techniques to identify the genetic changes present in the carcinogenic state, including DNA arrays, PCR, RT-PCR, gene sequencing, fluorescence in situ hybridization (FISH), etc. Contrarily, proteomic techniques make use of technologies including immunohistochemistry, enzyme-linked immunosorbent assay, and mass spectrometry to find novel cancer biomarkers and validate them in clinical trials [4]. Interleukin-8 (IL8) is a cytokine that has been linked to angiogenic and mitotic processes in various cancers. Researchers have shown that oral cancer patients had higher expression levels of IL8 in their saliva (720 pg mL^−1^ vs. 250 pg mL^−1^) than those in the control group [5]. However, there is an urgent need to create non-invasive, straightforward, and safe procedures for finding cancer biomarkers. In the disciplines of biomedical and diagnostic research, accurate disease-related biomarker detection is crucial [6]. Cancer biomarkers are proteins that are overexpressed in blood, saliva, and serum or present at low concentrations on the surface of cancer cells; biomarkers greatly aid in diagnosis [7,8]. Therefore, it is essential to create instruments capable of detecting biomarkers at such low quantities. Finding biomarkers can help with early disease diagnosis, disease staging, and therapy response monitoring. Numerous techniques have recently been created for evaluating cancer biomarkers [6]. Due to the antibodies’ strong affinity for the corresponding antigen, immunoassay is currently one of the most commonly used techniques for the detection of trace cancer biomarkers [9]. Tumor markers have been identified using a variety of immunoassay protocols, including chemiluminescence, fluorescence, Raman spectroscopy, electrochemistry, quartz crystal microbalance, and surface plasmon resonance [10,11]. Electrochemical immunoassays have garnered the most attention among these techniques because of their inherent benefits, including high sensitivity, cheap, low power requirements, and excellent compatibility with micromachining technologies [12,13,14,15]. Conventional immunoassays have often been used to find protein biomarkers. Enzyme-linked immunosorbent assays (ELISA), radio-immunoassays (RIA), and fluorescence, chemiluminescence, and mass spectrometric immunoassays are examples of immunological assays commonly used to detect cancer biomarkers. Conventional immunological assays, however, are challenging, labor-intensive, expensive, time-consuming, and unsuitable for point-of-care applications. As a result, point-of-care and research applications for protein measurement require straightforward, quick, sensitive, and affordable technologies [16,17,18,19,20].

Two-dimensional (2D) nanomaterials have received a lot of interest lately because of their unusual architectures and unique physical and chemical characteristics. Due to their behavior of accepting electrons and protons, layered transition metal dichalcogenides (TMDCs) are ideal for electrochemical sensor applications [21,22,23]. Transition metal dichalcogenides (TMDs) that resemble graphene have recently received a lot of academic attention due to their novel electrical conductivity features, optical capabilities, optoelectronic characteristics, and catalytic action [24]. Additionally, the exfoliated MoS_2_ condition can result in a large number of highly exposed soft-edge sites that are extremely active catalysts for electrochemical processes [25,26]. A type of layered substance resembling graphite is MoS_2_. This substance has attracted interest in a variety of fields, including electronic device creation, sensing, luminescence, and catalysis. Due to their significant surface area, minimal biotoxicity, remarkable biocompatibility, and anisotropic electron transportation, two-dimensional (2-D) films or flakes are used to detect molecular biomarkers as a result of the rapid advancements in material science. Additionally, certain MoS_2_-based proteins or DNA biosensors have been reported [27,28,29]. However, the bifunctional MoS_2_ nanocomposites based on MoS_2_ nanosheets frequently exhibit superior electrochemical behavior, catalytic performance, and sensing capabilities in addition to having stronger physical stability. MoS_2_ nanosheets have been viewed as viable options for determining the emerging concert of their unique sheet-like structure.

In comparison to current bio-sensing platforms, nanoparticles have a huge number of advantages, including narrow size distribution, effective surface modification, electron transport, electrochemical activities, quick electron transfer kinetics, desirable biocompatibility, and a high surface-to-volume ratio. The development of highly sensitive cancer biomarker sensors could make use of nanoparticles. Metal nanoparticles present significantly increased catalytic activity by acting in concert via a fast electron transfer reaction [30,31,32]. Moreover, because of the various elements that make up the electron coupling effect, nanoparticles can offer good stability, quick reaction, and high catalytic efficiency. Nanomaterials are used in immunoassays and sensors to provide: (i) a high surface area for antibody attachment and greater analytes access; (ii) signal amplification; and (iii) label-free real-time protein detection. As a result, the use of nanomaterials in the creation of sensors and assays will enable the ultrasensitive detection of very low amounts of cancer biomarkers. Nanomaterials have a strong affinity for cancer biomarkers, allowing for the biomarker's identification [33,34,35,36].

In the present work, we have demonstrated well-established salivary oral cancer biomarker interleukin-8 (IL8) detection using flower-structured molybdenum disulfide (MoS_2_)-decorated zinc oxide (ZnO) composite on GCE. The structural morphology and composition of the fabricated composite were then evaluated by different spectroscopic and microscopic analyses, such as transmission electron microscopy (TEM) and X-ray diffraction (XRD), as well as the differential pulse voltammetry (DPV) technique. Hence, the obtained results proved that the hybrid forms of metal oxide (ZnO) and molybdenum disulfide (MoS_2_) combination-based approach can be used in the clinical detection of IL8is, a cancer biomarker in multiple forms of cancer. For cancer biomarkers, the exfoliated MoS_2-_based composite is used in electrocatalysts for highly selective and sensitive electrochemical sensors. In addition, the developed sensor was used to detect biomarkers in real sample analysis.

## 2. Experimental

### 2.1. Chemicals and Reagents

Sodium molybdate dihydrate (Na_2_MoO_4_·2H_2_O), thiourea (H_2_NCSNH_2_), magnesium chloride (MgCl_2_), potassium chloride (KCl), sodium dihydrogen phosphate monohydrate (H_2_NaO_4_P·H_2_O), sodium phosphate dibasic heptahydrate (Na_2_HPO_4_·7H_2_O), sodium chloride (NaCl_2_), potassium hydroxide (KOH), zinc acetate dehydrate (Zn (CH_3_CO_2_)_2_·2H_2_O), acetone, ethanol, nitric acid (HNO_3_), and sulfuric acid (H_2_SO_4_) were acquired from Sigma-Aldrich. IL8 antigen and IL8 rabbit polyclonal antibodies were purchased from Bioss, Woburn, MA, USA. All chemicals were AR grade and used without any further purification. Ultrapure de-ionized water was used to prepare the necessary solutions. All other chemicals were purchased in analytical grade and used without further purification.

### 2.2. Synthesis of MoS_2_

The 2D MoS_2_ flower-like nano-flakes were successfully synthesized by a facile one-step hydrothermal technique. In total, 2.2 g Na_2_MoO_4_·2H_2_O and 2.0 g H_2_NCSNH_2_ were dissolved into 100 mL of de-ionized (DI) ammonia with strong stirring to form a homogeneous solution. After changing the pH assessment to less than 1 with 12 M HCl, the Mixed solution was shifted into a 250 mL Teflon-lined stainless-steel autoclave and hydrothermally treated for 18 h at 220 °C. The entire reaction method was cooled to ambient temperature. The final samples were then prepared for black precipitate centrifugation (9000 rpm), washing (water and ethanol), and drying for 12 h at 70 °C [37].

### 2.3. Preparation of MoS_2_/ZnO Composite

The flower-structured MoS_2_ nanosheet (50 mg) was exfoliated by sonication in water. After that, the synthesized ZnO-NPs (50 mg) were treated for 1 h under ultra-sonication to ensure good dispersion. Following dispersion, the solution was combined with a MoS_2_ suspension and left to stand at room temperature for 3 h with constant stirring. Following stirring, the product was collected, cleaned with DI water, and dried for 12 h at 80 °C in a hot air oven.

### 2.4. Electrode Preparation and Modification

**Scheme 1:**
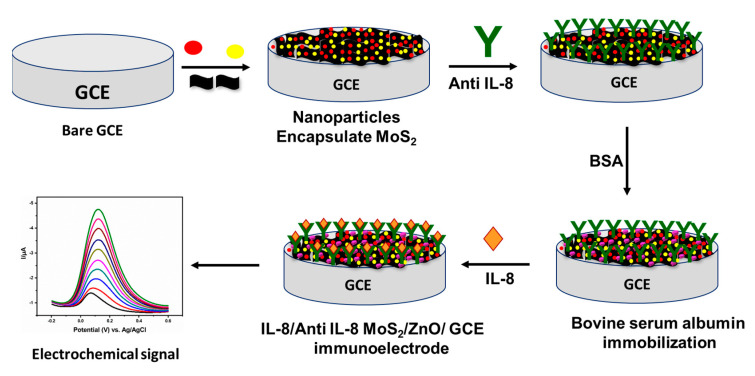
Schematic of Fabrication of MoS_2_/ZnO nanocomposite-based immunoelectrode for immunosensing application.

Using different grades of alumina slurry powder, a working glassy carbon electrode (GCE) with a surface area of 0.07 cm^2^ and diameter of 3 mm was polished to have a mirror-like surface (1.0, 0.05, and 0.3 microns). GCE was ultrasonically cleaned in water and ethanol numerous times for 10 min following polishing to eliminate any remaining surface debris. The GCE was then dried in a hot air oven at 50 °C before being used for further research. The GCE was then rinsed in de-ionized (DI) water. The MoS_2_/ZnO powder 0.01 g was dispersed in water and ultra-sonication for 1 h. After that, sonication 10 µL of dispersion solution was dropped on the cleaned GCE surface and dried overnight at room temperature (25 °C). It was thoroughly dried before being cleaned with de-ionized (DI) water and dried once more. A total of 20 L of newly made anti-IL8 antibodies were immobilized on the active film surface of the working electrodes for 4 h and then washed with 1× PBS buffer (pH 7.4) to remove any unwanted physical adsorption. Oral cancer biomarkers will be found using the immunosensors (Antibody/MoS_2_/ZnO modified GCE electrode) as they have been developed for the sensing of IL 8. The Fabrication of MoS_2_/ZnO nanocomposite-based immunoelectrode for immunosensing is shown in Scheme 1.

### 2.5. Determination of Human Saliva Real Sample Analysis

This fabricated immunosensor was put to the test by injecting a cancer biomarker into a controlled real sample of saliva. To remove any food particles or residues, the controlled real human saliva sample was centrifuged for 15 min at 8000 rpm. The clear supernatant was then collected for further use. This saliva sample will be spiked with various quantities of a cancer biomarker (without any dilution); the binding to an Ab/NPs adorned MoS_2_ modified GCEy electrode will then be observed using the detection of IL8 based on DPV method.

### 2.6. Electrochemical Measurements

All electrochemical experiments that used cyclic voltammeter (CV), differential pulse voltammetry (DPV) analysis, and amperometry (i-t) were conducted in a CHI 760C electrochemical workstation. All the measurements were performed under a conventional three-electrode electrochemical cell and assembled at room temperature. Platinum (Pt) and glassy carbon electrode (GCE) were used as the counter and working electrode, respectively, while Ag/AgCl (3 M KCl) was used as a reference electrode. A 0.1 M phosphate buffered saline solution (PBS pH 7.4) electrolyte was served as the electrolyte at room temperature for sensor applications.

### 2.7. Materials Characterization

The successful immobilization of Anti-IL8 onto AuNPs-rGO thin films was confirmed by XRD. X-ray diffraction (XRD) was used to analyze phase composition and crystalline structure measurements using an XRD, Bruker D8 Advance diffractometer consisting of Cu Kα radiation (λ = 1.541 Å). The morphological traits and nanostructures of the Mxene (Metal carbide) samples were further characterized by high solution transmission electron microscopy TEM (HR-TEM, JEM-2100 Plus, Jeol operating at 200 kV).

### 2.8. Analyte (IL8) Sample Preparation

By injecting IL8 into control saliva, this made-up immunosensor was evaluated for its ability to analyze genuine samples. To remove of any leftover food or residues, the control saliva sample was centrifuged for 15 min at 8000 rpm. The clear supernatant was then collected for future use. This saliva sample was spiked with various IL8 concentrations (without any dilution) and the binding on Anti-IL8/MoS_2_/ZnO/GCE immunoelectrodes was investigated using DPV.

In 10 mM PBS (pH 7.4), different concentrations of IL8 (from 500 fg/mL to 4500 fg/mL) were prepared and administered to the immunological electrode surface for 9 min. Utilizing the DPV approach, the immunoreactions that resulted were examined in a 1:1 *v*/*v* ratio of 10 mM PBS and 3 mM Zobell’s solution at a scan rate of 0.02 Vs^−1^, with pulse potential and pulse time values of 0.02 V and 0.07 s, respectively.

## 3. Results and Discussion

### 3.1. XRD Analysis

**Figure 1:**
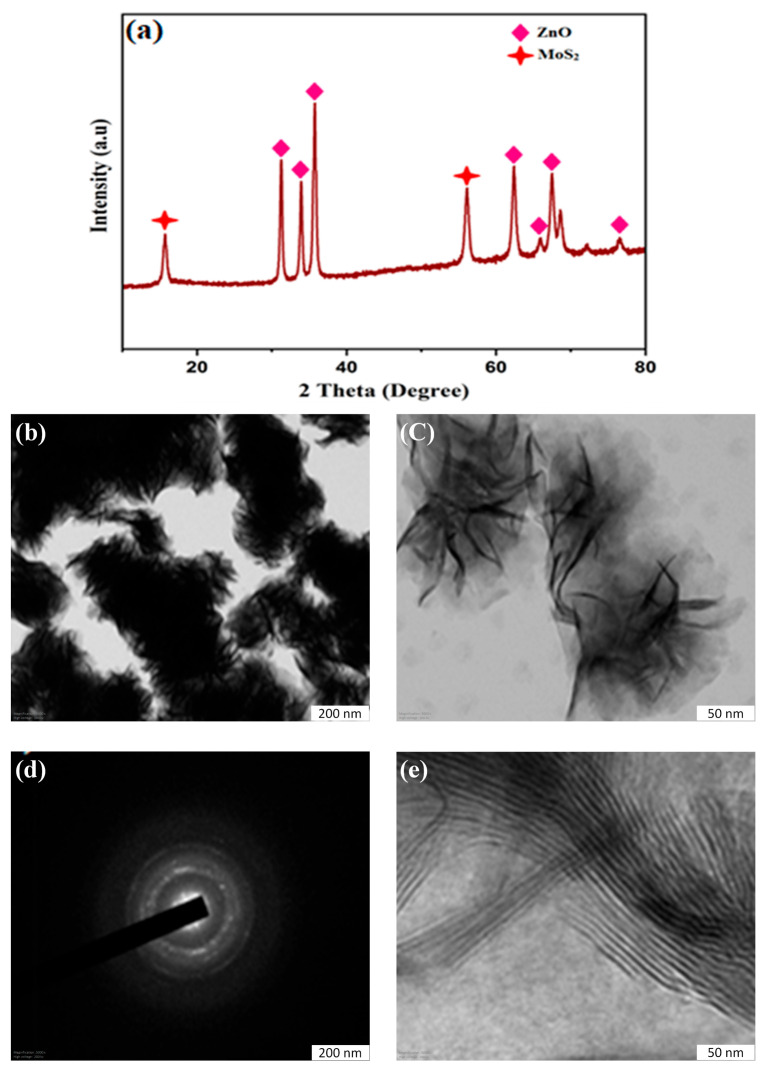
(**a**) XRD patterns of MoS_2_/ZnO nanocomposite. (**b**) HR-TEM image of exfoliated flower-structured MoS_2_ nanosheets. (**c**) HR-TEM image of flower-structured MoS_2_ nanosheets in high-resolution. (**d**) MoS_2_ nanosheets for selected area electron diffraction (SAED) pattern. (**e**) HR-TEM image of MoS_2_ lattice fringes.

The crystal structure and phase composition of the synthesized samples were identified and determined using powder XRD. The diffraction pattern of flower-shaped MoS_2_ is shown in Figure 1a, which reveals peaks at 2 = 13.88 and 42.61, respectively, that originate from the planes of the hexagonal 2H-MoS_2_ phase (JCPDS No. 37-1492). These peaks all supported the production of MoS_2_. The remaining peaks were observed at 2θ values of 31.42, 34.01, 35.88, 47.02, 56.22, 62.45, 65.75, 67.39, and 76.8, respectively, which corresponds to the crystal planes of the hexagonal ZnO nanoparticle’s structure (JCPDS card no. 36-1451). All these peaks support the formation of MoS_2_/ZnO nanocomposite [2,38]. Furthermore, no other phase segregation was detected in the XRD pattern; thus, the XRD results indicate the formation of a MoS_2_/ZnO nanocomposite.

### 3.2. HR-TEM and EDXS Analysis

**Figure 2:**
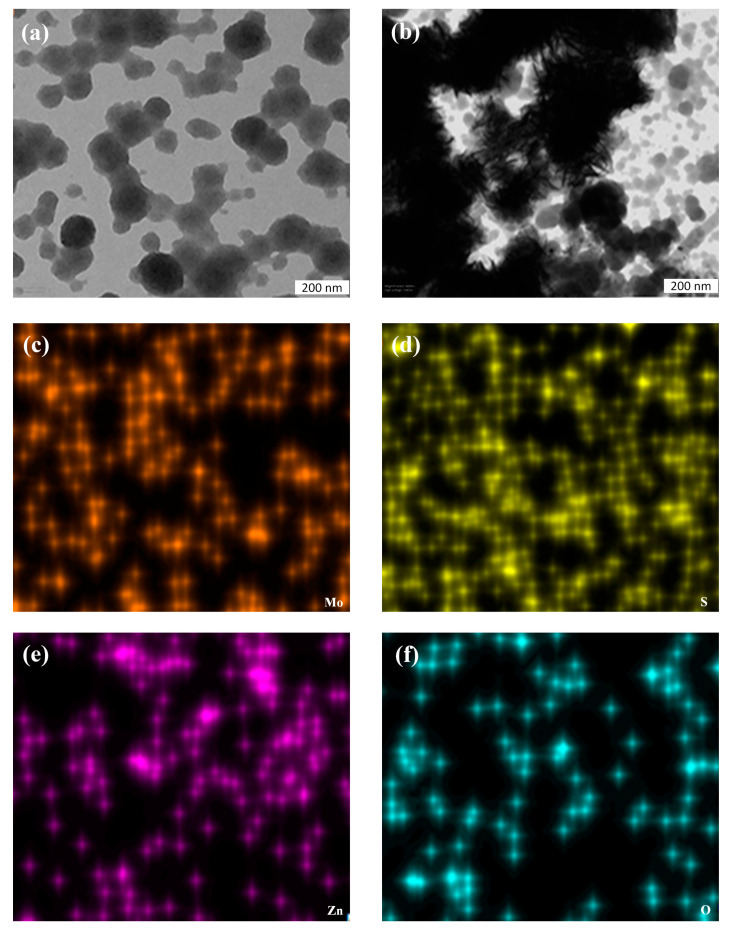
(**a**) HR-TEM images of synthesized ZnO; and (**b**) MoS_2_/ZnO nanocomposite. (**c**–**f**) EDAX spectrum of MoS_2_/ZnO nanocomposite.

The HR-TEM images of the as-prepared MoS_2_ flower structured morphology obtained (Figure 1b) are homogeneously distributed, which confirms the TEM images; we can see the flower-structured morphology assembled byMoS_2_, while the strong evidence for the formation of the flower structure of MoS_2_ was provided by HR-TEM analysis. Figure 1c shows a typical TEM image of MoS_2_ flowers, while HR-TEM measurement further confirms its nano sheet-like structure. Figure 2f clearly shows the ultrathin MoS_2_ nanosheet’s highly crystalline few-layer structure is well stacked with an interlayer distance observed as the (002) lattice planes of MoS_2_; the MoS_2_ nanosheets in HR-TEM image clearly indicate this fact. Figure 2c reveals the microspheres’ internal layer structure. Numerous nanosheets with a thickness of several nanometers may be seen to twist and spread out toward the spheres’ edges; the SAED pattern of MoS_2_ composite is shown in Figure 2d. The MoS_2_/ZnO nanocomposite TEM image, as shown in Figure 2b, is a flowered uniform MoS_2_ structure fully covered in ZnO. Figure 2 shows the EDXS spectrum of the MoS_2_/ZnO nanocomposite sample and confirms the presence of Mo, S, O, and Zn without any other impurities from the source ingredients. The HR-TEM images and analysis provided strong evidence for the formation of flower-structured MoS_2_ with a nano sheet-like structure, while the MoS_2_/ZnO nanocomposite was confirmed to contain Mo, S, O, and Zn without any other impurities through EDXS spectrum analysis [39].

### 3.3. DPV Detection of IL8

**Figure 3:**
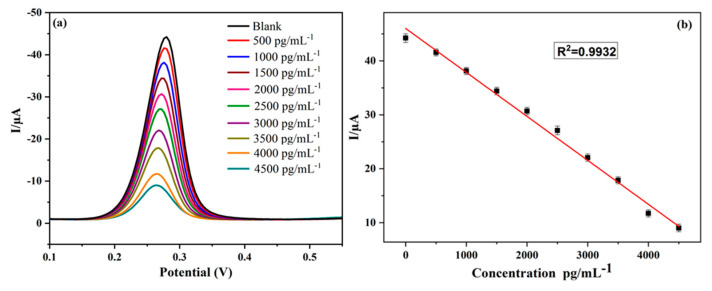
(**a**) Differential pulse voltammograms of Anti-IL8/MoS_2_/ZnO composite modified GCE electrodes recorded in different concentrations of IL8 range from 500 pg to 4500 pg. (**b**) A linear relationship between peak currents against concentrations of IL8.

This research demonstrated a fresh method for creating biosensors for the identification of bio-molecular signatures such as interleukin 8. Electrochemical detections have frequently used the highly sensitive DPV technique. With different concentrations of IL8 added to 0.1 M PBS (pH 7.4) buffer solution at a scan rate of 50 mV s^−1^, Figure 3a,b displays the typical DPV behaviors of the Anti-IL8/MoS_2_/ZnO nanocomposite modified electrode. Figure 3a shows the DPV curves for different concentrations of IL8on Anti-IL8/MoS_2_/ZnO modified GCE. As the concentration of analytes increases, the oxidation peak current also decreases linearly with a concentration range of 200–1400 μM. As a result of increased antigen binding, an insulating barrier has formed, preventing electron transport, which is the cause of the drop in current. The DPV current produced for the constructed sensor was proportional to the concentration of IL8 in the electrochemical cell. As is evident from the graph, the response current drops linearly as the concentration of the substrate (IL8) rises. With a linear correlation coefficient of R^2^ = 0.9960, Figure 3b demonstrates a strong linear relationship dependent on the concentrations of IL8 and the peak current response in the range of 200–1400 M. The experiment was repeated three times (*n* = 3) and the results are highly reproducible. The limit of detection is calculated from Anti-IL8/MoS_2_/ZnO modified electrode. This is a promising biosensor that could be used in the detection of IL8 at low concentrations. The detection limit prepared biological sensor of detection (LOD) is S/N = 3 of the biosensor; it was observed at 11.6 fM.

**Figure 4:**
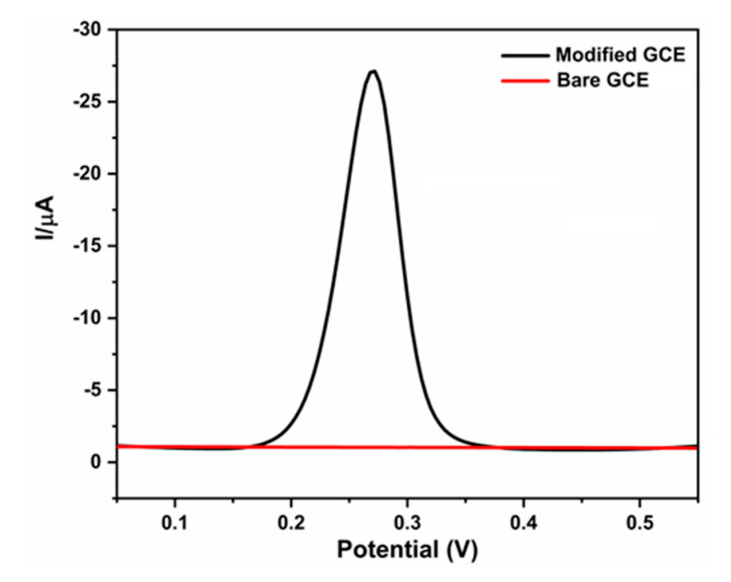
Differential pulse voltammograms of Anti-IL8/MoS_2_/ZnO composite modified GCE electrodes and bare GCE concentrations of IL8 range 2000 pg.

Electrochemical oxidation of 2000 pg IL8 was tested on a bare GCE and Anti-IL8/MoS2/ZnO composite modified GCE (at a scan rate of 50 mV s^−1^) in the potential range of 0.5–0.6 V, as shown in Figure 4. The Anti-IL8/MoS2/ZnO composite modified GCE exhibited a highly enhanced oxidation peak (black curve). In contrast, bare GCE showed no oxidation peak current (red curve). It was obvious that modified Anti-IL8/MoS2/ZnO composite produced a highly enhanced catalytic current of IL8. This study presents a promising biosensor using Anti-IL8/MoS2/ZnO nanocomposite modified electrode, which can detect IL8 at low concentrations with high sensitivity and reproducibility using DPV technique; this is demonstrated by the linear relationship between peak currents and concentrations of IL8 and the LOD of 11.6 fM [40].

### 3.4. Real Sample Analysis

Practical application of the proposed sensorofAnti-IL8/MoS_2_/ZnO modified electrode was tested for A Chin diluted human saliva; the real analysis sample was recorded in 0.1 M PBS, pH 7.4 using DPV technique via standard addition method. Saliva from a healthy individual was spiked with three different amounts to examine the effectiveness of the artificial immunosensor in real biological samples. Human saliva real sample analysis was collected from a healthy person at Medical College Hospital and Research Centre (SRM MCHRC), India. Human saliva sample was 1000 times diluted in 0.1 M PBS before testing. In order to determine the authentication of the proposed sensor, certain known concentrations of IL8100 nM, 200 nM, and 300 nM were spiked in the saliva sample and then detected (three measurements were taken in each sample). The recoveries were calculated from triplicate results. The results are provided in Table 1. Anti-IL8/MoS_2_/ZnO modified GCE showed good reproducibility and recovery rate for IL8 in real sample analysis. The experimental results of IL8 showed satisfactory recoveries that range between 101.5 and 103% in the blood–saliva sample. These results suggest that Anti-IL8/MoS_2_/ZnO modified GCE electrode is a promising candidate for real sample applications. The RSD Value shows less than 3%. In our present study, the results showed that the sensing platform shows effective performance in actual sample detection. The Anti-IL8/MoS_2_/ZnO modified electrode demonstrated excellent performance for the detection of interleukin 8 in diluted human saliva samples, with recoveries ranging from 101.5% to 103%, suggesting its potential as a practical biosensor for real sample analysis [41,42,43].

### 3.5. Anti-Interference of Coexisting Compounds

**Figure 5:**
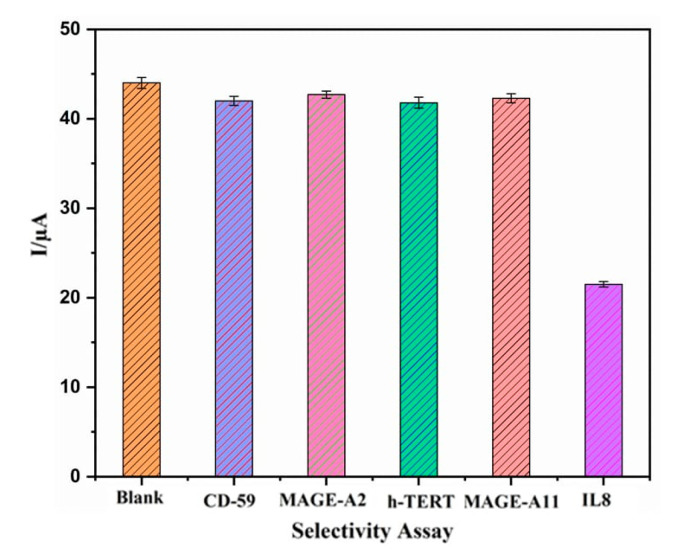
Bar graph showing the immunosensor response toward non-target analytes in comparison with response toward IL8.

During the anti-interference ability test, as depicted in Figure 5, we assessed whether the possible interfering species hTERT, S-100, MAGE-A2, and CD59, each at a concentration of 2000 pg/mL Anti-IL8/MoS_2_/ZnO composite modified GCE, were present. The addition of hTERT, S-100, MAGE-A2, and CD59 did not significantly alter the current response at DPV performance, while the analysis was maintained until the IL8 current was likewise reduced. This confirms the excellent specificity of the immunosensor for the IL8 antigen, as shown in Figure 5. These findings imply that the novel biosensor sensor system's IL8 detection is highly selective for the analytes without causing noticeable interference effects. The results show that the Anti-IL8/MoS_2_/ZnO composite modified GCE has excellent specificity for the IL8 antigen as it did not significantly alter the current response in the presence of possible interfering species hTERT, S-100, MAGE-A2, and CD59, confirming the biosensor's high selectivity for IL8 detection [44,45].

### 3.6. Repeatability and Reproducibility of Anti-IL8/MoS_2_/ZnO Modified Electrode

Repeatability and reproducibility are some of the most important indicators for the electrochemical sensor. All electrodes showed acceptable operational stability and no evident enzyme leaching. This might be a result of the significant electrostatic attraction between the MoS_2_/ZnO matrix and Anti-IL8 at the test pH value (7.4). Reproducibility of the proposed sensor of Anti-IL8/MoS_2_/ZnO modified GCE electrode was tested for IL8 in 0.1 M PBS using DPV technique by using a series of three consecutive measurements under the same conditions. It was found that the average relative standard deviations (RSDs) for the IL8 oxidation peak current were lower than 2.9%. The proposed sensor's excellent stability and the good reproducibility of the Anti-IL8/MoS_2_/ZnO modified electrodes are acceptable. We have also compared our analytical data with other reported methods for electrochemical detection of IL8 in Table 2. This proposed new method offers a number of advantages.

## 4. Conclusions

In summary, this work described Anti-IL8/MoS_2_/ZnO nanocomposite being successfully synthesized in constructing an electrochemical sensor for a sensitive system for the analyte without significant interference effects. The XRD, HR-TEM, and EDS mapping analysis were used to confirm the crystal structure, surface morphology, and elemental composition of the synthesized composite. The Anti-IL8/MoS_2_/ZnO composite outperforms other electrochemical sensors that have been previously reported because of its large specific surface area, enhanced mass and electron transfer, and high concentration of electroactive species. When compared to other analogs, these electrochemical sensors have good selectivity for IL8. In addition, the Anti-IL8/MoS_2_/ZnO composite has excellent reproducibility, while stability exhibited good sensitivity (1.257 fM) and anti-interference abilities, was enzyme-free, had wide detection range, and involved a simple process. This proposed biosensor work may offer new open doors for huge enhancements in the development of modified GCE. The fabricated immunosensor's high specificity, precision, and stability make it a promising platform for early-stage oral cancer detection; this method can be easily modified to detect other cancer types as it only requires samples from body fluids, such as saliva, blood, and urine, for cancer-specific biomarker detection.

## Figures and Tables

**Table 1:** Electrochemical detection of IL8 spiked in diluted human serum samples using Anti-IL8/MoS_2_/ZnO composite modified GCE as a sensor.

Added (fM)	Found (fM)	Recovery (%)	RSD (%) (*n* = 3)
10	10.2	102	1.92
20	20.6	103	2.36
30	30.5	101.6	2.24

**Table 2:** Various detection methods for IL-8 biomarkers.

Detection Method	Detection Range	Limit of Detection (LOD)	References
Chrono-coulometry	1 pg/mL to 1 μg/mL	~1 pg/mL	[46]
Cyclic voltammetry	0.1–10 pM	0.04 pM	[47]
Differential pulse voltammetry (DPV)	1 fg/mL–40 ng/mL	90 pg/mL	[47]
	100 fg/mL–5 ng/mL	51.53 ± 0.43 pg/mL	[48]
	500 fg/mL–4 ng/mL	72.73 ± 0.18 pg/mL	[40]
Anodic stripping voltammetry (ASV)	5–5000 fg/mL	3.36 fg/mL	[49]
Electrochemical impedance spectroscopy (EIS)	0.01–3 pg/mL	3.3 fg/mL	[50]
	900 fg/mL to 900 ng/mL	90 fg/mL	[51]
	0.02 pg/mL to 3 pg/mL	6 fg/mL	[44]
Amperometry	0.01–12.5 ng/mL	7.4 pg/mL	[52]
Amperometry	0.005–50 pM	3.9 fM	[53]
Amperometry	8–1000 pg/mL	8 pg/mL	[54]
Amperometry	10–1000 fg/mL	10 fg/mL	[55]
Amperometry	7–3750 fg/mL	7 fg/mL	[56]

## Data Availability

The data presented in this study are available in this article.
